# Assessing the Comparative Efficacy of Sentinel Lymph Node Detection Techniques in Vulvar Cancer: Protocol for a Systematic Review and Meta-Analysis

**DOI:** 10.3390/life14121538

**Published:** 2024-11-23

**Authors:** Balázs Vida, Balázs Lintner, Szabolcs Várbíró, Petra Merkely, Lotti Lúcia Lőczi, Nándor Ács, Richárd Tóth, Márton Keszthelyi

**Affiliations:** 1 Department of Obstetrics and Gynecology, Semmelweis University, 1082 Budapest, Hungary; vidabalazs.2000@gmail.com (B.V.); lintner.balazs.zoltan@semmelweis.hu (B.L.); merkely.petra@gmail.com (P.M.); keszthelyi.lotti.lucia@semmelweis.hu (L.L.L.); acs.nandor@semmelweis.hu (N.Á.); toth.richard@semmelweis.hu (R.T.); 2 Workgroup of Research Management, Doctoral School, Semmelweis University, 1085 Budapest, Hungary; varbiro.szabolcs@semmelweis.hu

**Keywords:** vulvar cancer, sentinel lymph node, Technetium-99m, Indocyanine green

## Abstract

This systematic review and meta-analysis protocol aims to evaluate the comparative efficacy of different sentinel lymph node (SLN) detection techniques in the management of vulvar cancer. Vulvar cancer, though rare, predominantly affects older women and requires effective management strategies. The SLN technique has become a standard approach for early-stage cases, offering reduced morbidity compared to complete lymphadenectomy. Currently, various SLN detection methods exist, including the use of Technetium-99m (Tc99m), Indocyanine Green (ICG), and superparamagnetic iron oxide (SPIO), but there is a lack of comprehensive comparison of their efficacy. This review will systematically search relevant databases, including PubMed, Scopus, Cochrane, Web of Science and Embase following PRISMA guidelines, to gather data from clinical trials. The primary outcome will be the detection rates of SLN techniques with secondary outcomes examining patient characteristics and procedural factors. The analysis will utilize random-effects models to compare detection rates across studies. The results of this study aim to provide insights into the optimal SLN detection method with potential implications for clinical practice guidelines in vulvar cancer management. The protocol is registered under the PROSPERO registration number CRD42024590774.

## 1. Introduction

Vulvar cancer is a rare malignancy comprising 5% of all gynecological cancers and 1% of all cancers in women. It predominantly affects patients between the ages of 65 and 75 with most cases diagnosed at an early stage [1,2]. The five-year survival rate varies significantly based on the stage at diagnosis, with early-stage (Stage I) disease offering a nearly 100% survival rate, while advanced-stage disease with lymph node metastasis reduces this figure to below 60% [2]. Prior to the introduction of sentinel lymph node (SLN) detection, complete lymphadenectomy (CL) was the standard practice. However, CL is associated with significant adverse effects (lymphocyst formation, lymphedema) that substantially impair patients’ quality of life. The development of a technique capable of identifying SLNs without the severe complications associated with CL was a pivotal advancement, leading to the evolution of the sentinel technique. According to the FIGO (Fédération Internationale de Gynécologie et d’Obstétrique) guidelines, for patients with squamous cell carcinoma, unifocal tumors smaller than 4 cm, and no clinical signs of groin node involvement, SLN detection and excision is recommended [3]. The current guidelines from the European Society of Gynecological Oncology (ESGO) mandate the use of a radiotracer, typically Technetium-99m (Tc99m), for SLN detection. These guidelines further recommend the combination of Tc99m with a colorimetric adjuvant, such as isosulfan or methylene blue dye, or an infrared imaging technique, such as ICG [4]. The success rate of SLN techniques can be affected by various factors. The earliest SLN detection methods employed blue dye, which was initially considered promising but was later surpassed by radiotracers, which offer significantly higher detection rates [5]. Tc99m is widely regarded as the gold standard in SLN detection for VC. However, Tc99m is not without its drawbacks. The requirement for a nuclear medicine facility, the need for administration around the tumor site the day before surgery, and the lack of real-time visual guidance pose logistical and practical challenges to its use [6]. A newer approach using ICG has shown promise in vulvar cancer [7]. ICG is a hydrophilic fluorescent agent that, when used with near-infrared (NIR) imaging, facilitates SLN biopsy in a single intraoperative step. Studies have demonstrated the feasibility and efficacy of ICG-guided SLN biopsy in early-stage VC [8]. Although ICG and NIR imaging have demonstrated comparable detection rates to Tc99m, the available data are often insufficient for definitive conclusions, and the supporting evidence remains limited [9,10,11]. An emerging approach showing promising detection rates and gaining increasing attention in vulvar cancer management is the superparamagnetic iron oxide (SPIO) method, which eliminates the need for radioactive tracers, provides high detection accuracy, and offers logistical advantages by allowing same-day procedures without the requirement for specialized nuclear medicine facilities [12,13]. Recognizing the need for a comprehensive evaluation, this study aims to conduct a systematic review comparing the detection rates of all currently used SLN techniques. By addressing this gap in the literature, we seek to provide critical insights into the comparative efficacy of these methods in the management of vulvar cancer.

## 2. Experimental Design

This systematic review will be conducted and reported following the Preferred Reporting Items for Systematic Reviews and Meta-Analyses (PRISMA) guidelines.

### 2.1. Eligibility Criteria

#### 2.1.1. Type of Studies

We will include prospective and retrospective clinical trials, as well as randomized controlled trials, without any language restrictions. The search will cover all relevant studies available in the databases from their inception up to 15 August 2024.

#### 2.1.2. Types of Participants

Eligible studies will include women diagnosed with vulvar cancer classified as FIGO stage T1a or T2, where the tumor size exceeds 2 cm but remains under 4 cm, with an invasion depth greater than 1 mm, and without detectable lymph node metastasis. While vulvar cancer predominantly affects older women, participants of all age groups will be included in the analysis.

#### 2.1.3. Type of Methods

We aim to include all currently available SLN detection methods. These will include the Tc99m isotope, blue dye, ICG, and SPIO solution.

#### 2.1.4. Type of Outcomes

The primary outcome of interest will be the detection rates (DRs). Secondary outcomes will focus on patient-specific factors such as mean and median age (year), body mass index (BMI) (kg/m^2^), tumor size (cm) and grade, tumor position (lateral, medial) and anatomical location (distance to the midline/clitoris/anus/vagina/urethra (cm)), type of surgery (wide local excision, hemivulvectomy, vulvectomy), lymph node assessment (unilateral vs. bilateral), lymphovascular space invasion (LVSI) (1–4), hystotype (carcinoma vs. melanoma) and their influence on detection rates. Additionally, we aim to assess procedure-related variables, such as the doses (activity, mg) and concentrations (mg/mL) of substances used, type of surgery, and short and long-term complications.

### 2.2. Materials and Equipment

Our search will utilize the terms “sentinel” and “vulv” across the most comprehensive online databases, including PubMed, Scopus, Cochrane, Embase, Web of Science, and Medline. The search will cover all records available up to and including 15 August 2024.

### 2.3. Detailed Procedure

We will follow the recommendations of the Cochrane Collaboration for planning, study selection, and data extraction. All relevant studies will be identified through our search strategy across the specified databases. Duplicate records will be removed using the bibliographic management tool EndNote, X 9 version: 21.4 and Rayyan will be utilized for the screening and selection process. The initial screening will be conducted based on titles and abstracts, which are followed by full-text reviews. Additional relevant articles will be manually identified by reviewing the reference lists of the included studies. Two independent reviewers will be responsible for the selection and data extraction, using a predefined standardized data collection form. Any disagreements will be resolved with the involvement of a third independent reviewer. Cohen’s kappa coefficient will be calculated to assess inter-rater reliability during both the selection and extraction phases.

### 2.4. Data Items

The extracted information will include the date, name of the author, country, type of malignancy, study design, patient number and groin number, detection rates, doses and concentrations, mean age, and the mean BMI of the patients. If any data are missing, incomplete, or unclear, the authors will be contacted for clarification.

### 2.5. Outcomes and Prioritization

Our primary outcome will be the DR of each SLN detection method, including combinations of different techniques, with a focus on comparing their effectiveness. A secondary objective will be to investigate the relationship between detection rates and patient characteristics, such as average BMI. Additionally, if sufficient data are available, we will attempt to gather information on the doses and concentrations of the substances used in these procedures.

### 2.6. Effect Measures

For dichotomous outcomes, such as successful SLN detection or adverse events, we will report risk ratios (RRs) along with risk differences (RDs) and group proportions for better clarity. For continuous variables, including patient-related data (e.g., BMI), we will present mean differences (MDs) or differences between medians (MedDs), depending on the format in which the data are provided in the studies. We will also report the group-specific means or medians to enhance the interpretability of the results.

### 2.7. Risk of Bias

Two members of the review team will assess the risk of bias via the robvis visualization tool for all of the studies. If a disagreement arises, a third reviewer will be involved. The tool evaluates five areas of potential bias: bias from the randomization process, bias due to deviations from the intended interventions, bias from missing outcome data, bias in outcome measurement, and publication bias: bias in the selection of reported results. Each domain includes signaling questions designed to gather relevant information. The answers to these questions are processed through algorithms to rate each domain as having a low risk of bias, some concerns, or a high risk of bias. These individual domain ratings are then combined into an overall risk-of-bias judgment, which also falls into the categories of low risk, some concerns, or high risk of bias.

### 2.8. Data Synthesis

If the included studies and their results show sufficient homogeneity, we will carry out a meta-analysis using a random-effects model. Both qualitative and quantitative data synthesis will be undertaken. At least three studies will be required to conduct a meta-analysis. The random effects model will be employed to combine effect sizes using a frequentist approach. For dichotomous outcomes, we will calculate risk ratios (RRs) along with 95% confidence intervals (CIs) as the effect size measure. Proportions of events of interest will be pooled separately for each group. For continuous variables, we will use either the mean difference (MD) or the difference between medians (MedD) with 95% CI as the effect size, depending on how the data are presented. If only quartiles are provided, we will estimate the mean and standard deviation (SD) assuming the distribution is either normal or lognormal; otherwise, the median differences will be pooled. Pooled RR based on raw data will be calculated using the Mantel–Haenszel method, with the exact Mantel–Haenszel technique applied to manage zero cell counts. The inverse variance weighting method will be utilized for calculating the pooled RR and MD when raw data are not available. To enhance precision, we will adjust the pooled confidence intervals using the Hartung–Knapp method when it produces a more conservative result. The heterogeneity variance (τ^2^) will be estimated using the restricted maximum-likelihood estimator, and the confidence interval will be derived using the Q profile method. Heterogeneity among the studies will be evaluated using Higgins and Thompson’s I^2^ statistic. Results will be deemed statistically significant if the confidence interval excludes the null value. The findings will be visually represented using forest plots, and prediction intervals will be reported when applicable. We will explore model-fitting parameters and identify potential outliers using various influence measures and visual plots. All statistical analyses will be performed using the R software package (R Core Team) version: 4.4.2. The confidence in effect estimates for each reported outcome will be assessed using the Grading of Recommendations, Assessment, Development, and Evaluation approach by two reviewers, and possible disagreement will be assessed by a third reviewer.

### 2.9. Ethical Concerns

Patients or members of the public will not participate in our research; therefore, ethical approval is not required for this study, as no original data will be collected

### 2.10. Registration

Prospero registration number: CRD42024590774

## 3. Expected Results

While previous meta-analyses and systematic reviews have demonstrated the efficacy of ICG in SLN detection, a direct comparison with the ESGO-recommended tracer, Tc99m, remains necessary and warrants an updated analysis. Additionally, the novel SPIO method, given its promising potential, and blue dye, one of the earlier techniques still in use, require further evaluation and comparison against established methods. By employing the RoB 2.0 tool and robvis visualization software, our study strives to minimize bias and ensure a high level of evidence quality. The findings of this systematic review may influence future clinical guidelines, as it aims to provide an up-to-date comparison of all currently employed SLN detection techniques in vulvar cancer. However, the robustness of our conclusions may be limited by the quality of the individual studies included, particularly if there are gaps or inconsistencies in the reported data.

## Data Availability

Due to this article’s nature, no original data were used.
